# Predicting Caregiver Anxiety and Depression From Patient Distress in Brain Tumor Dyads: Actor‐Partner Interdependence Model

**DOI:** 10.1002/cam4.71271

**Published:** 2025-09-29

**Authors:** Anna‐Maria Kisić, Maike K. Klett, Ralf Schaefer, Caterina Quente, Michael Sabel, Marion Rapp, André Karger

**Affiliations:** ^1^ Clinical Institute of Psychosomatic Medicine and Psychotherapy Medical Faculty and University Hospital Düsseldorf, Heinrich Heine University Düsseldorf Düsseldorf Germany; ^2^ Center for Integrated Oncology Aachen Bonn Cologne Düsseldorf (CIO ABCD) Düsseldorf Germany; ^3^ Department of Neurosurgery Medical Faculty and University Hospital Düsseldorf, Heinrich Heine University Düsseldorf Düsseldorf Germany

**Keywords:** anxiety, brain neoplasms, cancer, caregiver burden, depression, oncology, psychological support, psycho‐oncology

## Abstract

**Objective:**

Malignant brain tumors place significant physical, cognitive, and emotional strain on patients and caregivers. Psychosocial distress screening is part of standard care for patients, while caregiver screening remains challenging. This study examined the association of patient psychosocial distress at diagnosis with caregiver anxiety and depression over time.

**Methods:**

This secondary analysis used data from a prospective, single‐center, observational study of malignant brain tumor dyads. To assess the association of patient psychosocial distress at diagnosis (T0) with caregiver anxiety and depression at T0 and at 3 (T1) and 6 (T2) months post‐diagnosis, the Actor‐Partner Interdependence Model (APIM) was used.

**Results:**

Complete data from 58 dyads were included at T0, 43 at T1, and 41 at T2. Patient distress at T0 predicted caregiver depression at T1 (*β* = 0.310, *p* = 0.007) and T2 (*β* = 0.322, *p* = 0.005), and caregiver anxiety at T2 (*β* = 0.303, *p* = 0.020). Caregiver distress at T0 did not predict patient anxiety and depression at any time point. For both patients and caregivers, distress at T0 predicted their own anxiety and depression at T0 and their anxiety at T1. For caregivers, distress at diagnosis also predicted anxiety at T2.

**Conclusions:**

Psychosocial distress experienced by patients with malignant brain tumors at diagnosis significantly predicts their caregivers' anxiety and depression over time. Caregivers at risk of increased anxiety and depression could therefore be identified by screening for patient distress. These findings also highlight the critical need for early psychosocial support for both patients and caregivers.

**Trial Registration:**

Retrospectively registered in the German Clinical Trial Register (10 July 2024; DRKS00034637)

## 1 Background

Malignant brain tumors are linked to a poor prognosis, with an estimated 10‐year relative survival rate of 29.3% for primary brain tumors [1]. Patients require intensive treatments that significantly impact their quality of life [2, 3]. As the disease progresses, debilitating symptoms such as personality changes, neurocognitive impairment, and functional decline occur, causing a high psychological burden for patients [4, 5, 6].

Caregivers represent an important psychosocial resource for the patient, particularly in cases of severe illnesses like brain tumors. The physical and neuropsychological symptoms experienced by patients, however, also affect the quality of life of caregivers, placing increasing demands on them [4, 7, 8, 9]. Caregivers of patients with brain tumors have been reported to experience significant psychosocial and physical burden, even when compared with caregivers of other cancer groups [4, 10, 11, 12]. Furthermore, psychosocial factors such as social support, financial toxicity, and socioeconomic status affect the distress and well‐being of cancer patients and their caregivers [13, 14, 15]. In a study by Loughan, Reid [16], patients with primary brain tumors reported themselves that their caregivers were faced with a multitude of responsibilities (e.g., managing medical care, financial responsibility, handling emotional distress).

Although distress screening is standard practice for oncology patients [17, 18], few routine programs exist to screen for caregiver distress [19]. Screening for caregiver psychosocial distress poses several challenges in clinical care, including logistical difficulties in reaching the caregivers, a lack of standardized and widely used instruments, and a lack of resources [20]. Furthermore, Reblin and Small [13] showed that early burden in caregivers of patients with primary brain tumors can predict later psychological distress. Therefore, early and tailored support for caregivers is essential to prevent further decline in quality of life alongside patient care [12, 21].

Patient and caregiver distress are closely interrelated. In pancreatic cancer, patient distress has been shown to be a significant predictor of concurrent caregiver distress, anxiety, depression, and perceived burden [22]. In dyads of patients with primary brain tumors and their caregivers, Braun, Aslanzadeh [23] provided the first evidence of dyadic effects related to fear of cancer recurrence. Surprisingly, caregiver depressive symptoms and death anxiety negatively predicted patients' fear of cancer recurrence. The authors referred to the Dyadic Stress Model [24], suggesting that compensatory dynamics could explain these findings [23]. According to this model, distress within couples may fluctuate in a complementary manner: when one partner experiences higher distress, the other may compensate by adopting more active coping strategies or providing support. In caregivers of patients with malignant glioma, distress was shown to be influenced by the patient's depressive symptoms and age [25]. While direct caregiver screening represents the best practice, caregivers with increased distress and support needs could be identified through the distress screening of their respective partners affected by cancer until implementation barriers are overcome. However, given the cross‐sectional design of both studies, research on the longitudinal interrelation between psychosocial distress in patients and their caregivers is lacking, especially for brain tumor dyads.

The aim of this secondary analysis was to examine the association between psychosocial distress in patients with brain tumors at diagnosis and anxiety and depression in their respective caregivers over time. We focused on the dyads as the unit of analysis and accounted for the interdependent nature of patient‐caregiver dyads using the Actor‐Partner Interdependence Model (APIM). We hypothesized that patient psychosocial distress at diagnosis was significantly associated with caregiver anxiety and depression at (1) diagnosis, (2) after 3 months, and (3) after 6 months (partner effects). As secondary aims, we explored the association of both patients' and caregivers' psychosocial distress scores with their own anxiety and depression (actor effects) as well as the association of caregiver distress with patient anxiety, and depression (partner effects) over all time points.

## 2 Methods

### 2.1 Study Design

The present secondary analysis was conducted using the longitudinal data from a prospective, observational, monocentric study that recruited patients with malignant brain tumors and their primary caregivers (patient‐caregiver dyads). The main study is described in detail by Karger, Kisić [26]. The study was registered in the German Clinical Trials Register (DRKS00034637) and approved by the ethics committee of the medical faculty at Heinrich Heine University Düsseldorf (ID: 2018‐338‐ProspDEuA). During the study, participants gave written informed consent and were able to withdraw from the study at any time.

This secondary analysis is reported in accordance with the Strengthening the Reporting of Observational Studies in Epidemiology (STROBE) Statement [27].

### 2.2 Setting

**FIGURE 1:**
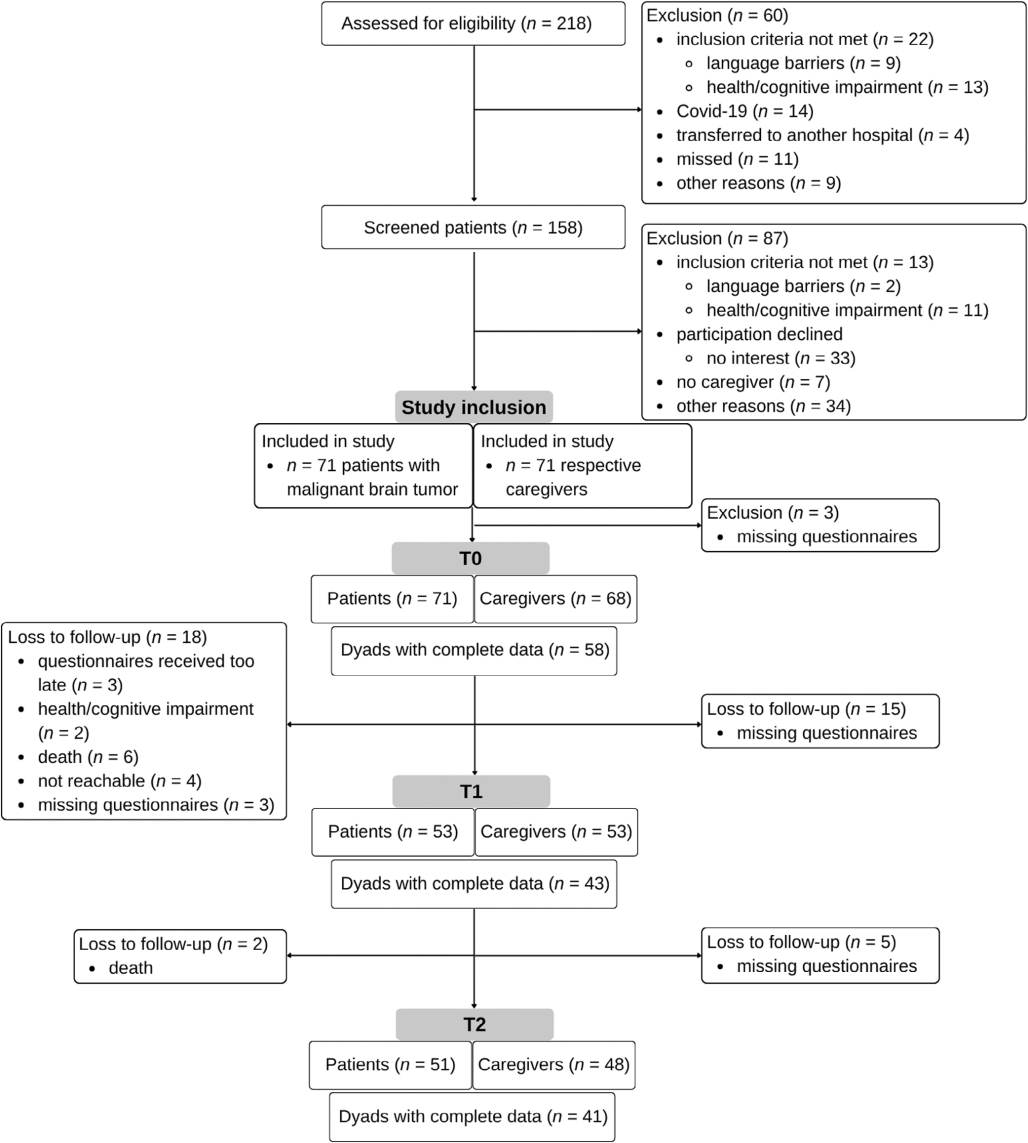
Flow chart.

Recruitment for the primary study took place from July 2019 to August 2020 at the Department of Neurosurgery, University Hospital Düsseldorf. The Department of Neurosurgery provided the study team with a list of all patients undergoing brain tumor surgery. All reachable patients were approached by the study team in the hospital ward 3 to 7 days after surgery, informed about the study, and checked for inclusion criteria. A detailed flow chart is shown in Figure 1. If consent was obtained, patients with malignant brain tumors and their caregivers completed self‐report questionnaires for psychosocial distress, anxiety, depression, fear of cancer progression, and quality of life 3 to 7 days (T0), 3 months (T1), and 6 months (T2) post‐diagnosis. If the caregiver was not available during the inpatient visit, the caregiver received information about the study by post and was invited to participate if interested. The assessment time points were chosen according to the regularly scheduled visits during treatment. The sociodemographic and medical data were extracted from the medical records and partially collected at T0.

### 2.3 Participants and Procedures

Inclusion criteria for the main study were (1) first diagnosis of a malignant brain tumor, including both primary and secondary malignant brain tumors; (2) elective admission for primary neurosurgery; (3) age over 18 years; (4) legal capacity; (5) ongoing adjuvant treatment at the Department of Neurosurgery; and (6) sufficient German proficiency for questionnaire assessment. Exclusion criteria were (1) severe aphasic disorder; (2) terminal palliative care with a life expectancy of < 3 months; and (3) physical or cognitive impairments preventing participants from study completion. The patients themselves identified their primary caregiver. Caregivers were defined as “primary care providers”, meaning the person who primarily cares for the patient during their illness [28].

### 2.4 Variables

#### 2.4.1 Psychosocial Distress

The National Comprehensive Cancer Network (NCCN) Distress‐Thermometer (DT; [29]) is a single‐item visual analog scale that measures psychosocial distress. The scale ranges from 0 to 10, with higher scores indicating more distress and a score of ≥ 5 indicating increased distress [29]. Validity and reliability for the German version have been confirmed [29].

#### 2.4.2 Depression and Anxiety

Anxiety and depression were assessed using the Hospital Anxiety Depression Scale (HADS) [30], which includes seven items each for anxiety and depression. The four‐point Likert scales range from 0 to 3, with a higher total score indicating more symptoms. A cut‐off score of ≥ 8 for each subscale indicates clinically relevant anxiety or depression [31]. The psychometric validation of the scale has been extended to cancer patients [32].

#### 2.4.3 Sociodemographic and Medical Characteristics

We assessed age, sex, housing situation, citizenship, country of birth, marital/relationship status, relationship to the patient or caregiver, children, education, employment, psychotropic drug intake, psychotherapy, date of diagnosis, type of cancer diagnosis, treatment, and disease progression in self‐report questionnaires. The localization of the tumor and the functional status (ECOG) [33] were extracted from the patient's medical records.

### 2.5 Statistical Analysis

Descriptive statistics were presented using frequencies and percentages for categorical data. To examine potential differences in differential sociodemographic and clinical characteristics between completers and dropouts, dropout analyses were conducted using *t*‐tests and Chi‐squared tests. Continuous data were described by means (*M*) and standard deviations (SD). As patients and caregivers were dyads, a dependency between the data could be assumed. Therefore, APIMs [34, 35] were performed to account for this interdependence. The APIM is a model of dyadic relationships that integrates a conceptual view of interdependence with appropriate statistical techniques for measuring and testing this interrelationship (for a detailed overview: [34]). The APIM assumes that an individual's outcome score depends not only on his/her individual score on the predictor variable but also on his/her partner's score. Thus, the APIM estimates actor and partner effects. Actor effects represent the influence of an individual's own distress score on the same individual's outcome variable (anxiety, depression; intraindividual). Partner effects represent the influence of an individual's own distress on his or her partner's outcome variable (anxiety, depression; interindividual). As APIMs are based on complete datasets, the same number of cases for both the predictor and outcome variables were included in the analyses. An online multilevel modeling tool was used for all APIM analyses. The online tool was developed using the R package “lavaan” [36]. The analyses were based on structural equation models with maximum likelihood estimations. The tests of coefficients within the APIMs were *Z*‐tests. Partial correlations between predictor and outcome variables reflect effect sizes. The remaining analyses were performed using R statistical software (Version 4.3.1) [37]. An alpha level of 0.05 was considered statistically significant.

## 3 Results

### 3.1 Participants

Two hundred and eighteen patients with primary or secondary malignant brain tumors were potentially eligible for study participation. A total of 71 patients and their caregivers were recruited (see Figure 1). Since only complete data can be used for the APIM, this resulted in 58 dyads for time T0, 43 dyads for T1, and 41 dyads for T2.

In most patients (*n* = 40, 69.0%), a glioblastoma (primary brain tumor) was diagnosed, while in 18 patients (31.0%), cerebral metastases (secondary brain tumor) were identified. All patients were undergoing systemic and/or local therapy during the study period. Caregivers were predominantly partners, followed by family members. Detailed sociodemographic and clinical data are summarized in Table 1.

**TABLE 1:** Demographic and disease‐related data from patients and their caregivers at T0.

	Patient (*n* = 58)		Caregiver (*n* = 58)	
	*n* (missing)	%	*n* (missing)	%
Age	58 (0)	100 (0)	58 (0)	100 (0)
Mean; SD	55.2	15.1	53.3	15.2
Sex	58 (0)	100 (0)	58 (0)	100 (0)
Women	28	48.3	36	62.1
Men	30	51.7	22	37.9
Housing situation	58 (0)	100 (0)	58 (0)	100 (0)
Same household	40	69.0	39	67.2
Other household	7	12.1	14	24.1
Alone	11	18.9	5	8.7
German citizenship	58 (0)	100 (0)	58 (0)	100 (0)
Yes	48	82.8	48	82.8
Relationship status	58 (0)	100 (0)	58 (0)	100 (0)
Married, in partnership	45	77.6	51	87.9
Unmarried, widowed	13	22.4	7	12.1
Children	58 (0)	100 (0)	58 (0)	100 (0)
Yes	38	65.5	37	63.8
< 18 years	12	20.7	13	22.4
≥ 18 years	26	44.8	24	41.4
Educational level	58 (0)	100 (0)	58 (0)	100 (0)
< 12 years	32	55.2	33	56.9
≥ 12 years	26	44.8	25	43.1
Employment	56 (2)	96.6 (3.4)	58 (0)	100 (0)
Working	23	39.7	35	60.3
Retired	21	36.2	19	32.8
Not working	12	20.7	4	6.9
Relationship with patient/caregiver	58 (0)	100 (0)	58 (0)	100 (0)
Partner	38	65.5	38	65.5
Family member	16	27.6	16	27.6
Other (e.g., friend)	4	6.9	4	6.9
Psychotropic drug intake	57 (1)	98.3 (1.7)	52 (6)	89.7 (10.3)
Yes	9	15.5	5	8.6
No	48	82.8	47	81.1
Psychotherapy (current/previous)	52 (6)	89.7 (10.3)	52 (6)	89.7 (10.3)
Yes	11	19.0	11	19.0
No	41	70.7	41	70.7
Functional status	58 (0)	100 (0)		
ECOG 0–2	56	96.6		
ECOG 3–4	2	3.4		
Diagnosis	58 (0)	100 (0)		
Primary brain tumor	40	69.0		
WHO II	8	20.0		
WHO III	6	15.0		
WHO IV	24	60.0		
Unknown	2	5.0		
Metastasis	18	31.0		

*Note:* WHO II includes oligodendroglioma and diffuse astrocytoma. WHO III includes anaplastic glioma, anaplastic astrocytoma, and anaplastic oligodendroglioma. WHO IV includes glioblastoma and diffuse midline glioma.

Abbreviations: ECOG, Eastern Cooperative Oncology Group; SD, standard deviation; WHO, World Health Organization.

Dropout analyses revealed no significant differences in baseline distress, baseline depression, age (all *t*(56) ≤ 1.24, all *p* > 0.221), sex, or having children (all *χ* ^2^(1) ≤ 2.56, all *p* > 0.109) between completers and dropouts. However, patients who dropped out reported significantly lower anxiety at baseline (*M* = 3.24, SD = 2.59) compared to completers (*M* = 6.93, SD = 5.04), *t*(56) = 2.86, *p* = 0.006.

### 3.2 Descriptive Data

Descriptively, caregivers indicated higher distress, anxiety, and depression scores than patients at all time points (see Table 2).

**TABLE 2:** Means and standard deviations for patient and caregiver outcomes.

	T0 (*n* = 58)		T1 (*n* = 43)		T2 (*n* = 41)	
	Patients	Caregivers	Patients	Caregivers	Patients	Caregivers
DT						
Mean	5.43	6.86	5.11	5.98	4.90	5.76
SD	2.86	2.32	2.75	2.48	2.84	2.61
HADS‐A						
Mean	5.84	9.24	6.84	9.40	6.20	8.27
SD	4.75	4.86	4.34	4.33	3.69	3.94
HADS‐D						
Mean	5.01	6.48	6.42	7.21	5.78	6.27
SD	4.51	3.89	5.55	4.52	4.59	3.98

*Note:* The reported sample sizes (*n*) include patients with complete baseline (T0) distress data and complete anxiety/depression data at the respective time point.

Abbreviations: DT, NCCN distress thermometer; HADS‐A, Hospital Anxiety and Depression Scale‐Anxiety; HADS‐D, Hospital Anxiety and Depression Scale‐Depression; SD, standard deviation.

### 3.3 Association of Psychosocial Distress at Diagnosis with Anxiety Over Time

#### 3.3.1 Partner Effects

**FIGURE 2:**
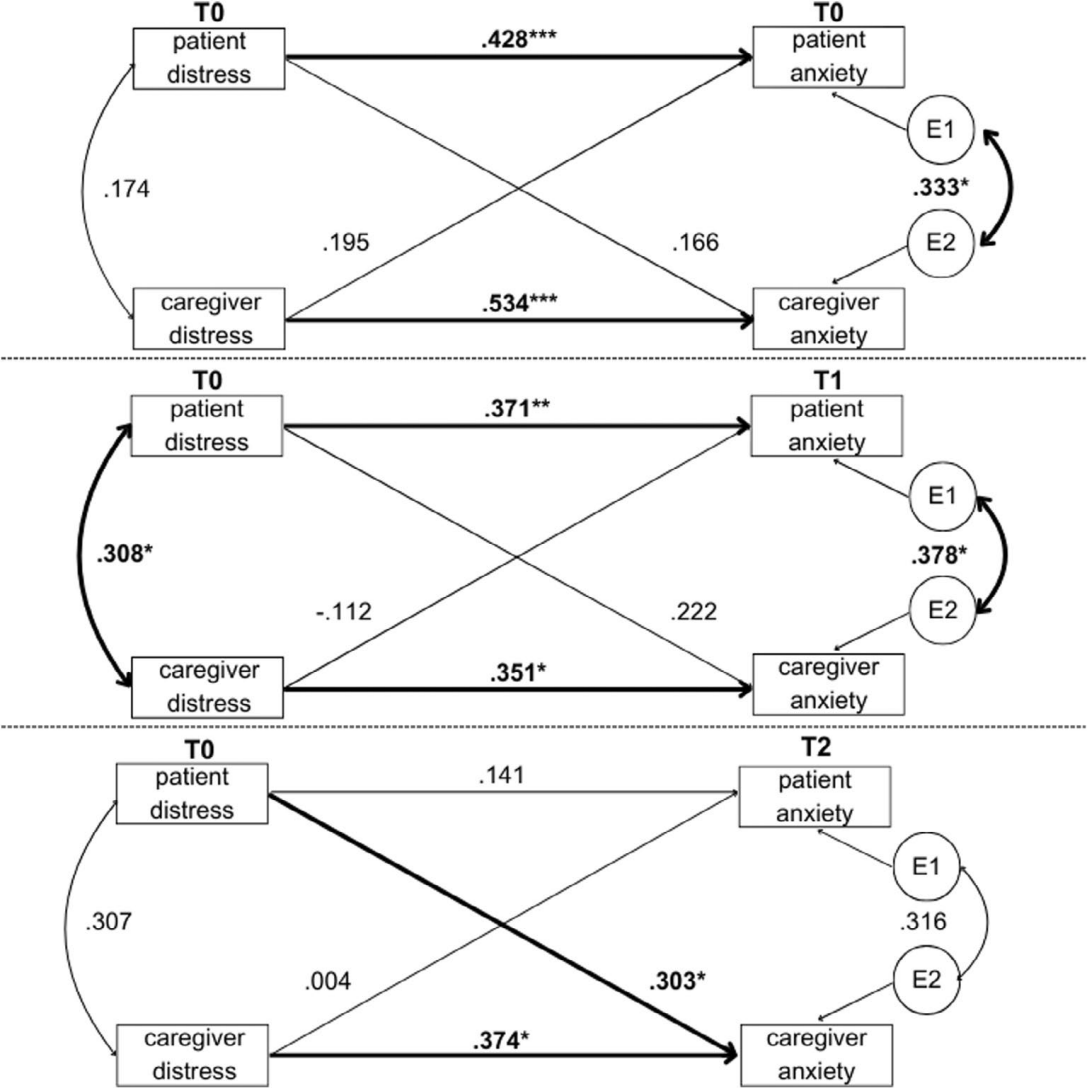
Actor partner interdependence model of anxiety over time. Values represent standardized beta coefficients. E1 = residual/error term for patient, E2 = residual/error term for caregiver. T0 = Baseline, T1 = 3 months post‐diagnosis, T2 = 6 months post‐diagnosis. Number of included dyads for each analysis: T0: *N* = 58 dyads, T1: *N* = 43 dyads, T2: *N* = 41 dyads. *p < 0.05, **p < 0.01, ***p < 0.001.

No significant partner effects on anxiety were found at diagnosis (T0). The patients' psychosocial distress scores did not influence the caregivers' anxiety scores at T0 (*p* = 0.111, *r* = 0.21), nor were caregivers' distress scores associated with patients' anxiety scores (*p* = 0.118, *r* = 0.20). After 3 months (T1), the same pattern was observed. The patients' distress scores at T0 did not significantly affect the caregivers' anxiety scores at T1 (*p* = 0.083, *r* = 0.26). Likewise, there was no significant association between caregivers' distress scores and patients' anxiety scores (*p* = 0.513, *r* = −0.10). At T2 (after 6 months), a significant partner effect could be observed, with patients' distress scores at T0 significantly influencing caregivers' anxiety scores at T2 (*p* = 0.020, *r* = 0.34). No significant association was identified between caregivers' distress scores at T0 and patients' anxiety scores at T2 (*p* = 0.980, *r* = 0.004). A detailed representation of the model is shown in Figure 2.

#### 3.3.2 Actor Effects

At T0, the individual's psychosocial distress score was significantly associated with his/her own anxiety score for both patients and caregivers (patients: *p* < 0.001, *r* = 0.49; caregivers: *p* < 0.001, *r* = 0.48). The same pattern was observed at T1. There was a significant association between an individual's distress score at T0 and their own anxiety at T1 (patients: *p* = 0.005, *r* = 0.39; caregivers: *p* = 0.034, *r* = 0.31). At T2, this intraindividual association remained significant only for caregivers (patients: *p* = 0.318, *r* = 0.15; caregivers: *p* = 0.025, *r* = 0.33).

### 3.4 Association of Psychosocial Distress at Diagnosis with Depression Over Time

#### 3.4.1 Partner Effects

**FIGURE 3:**
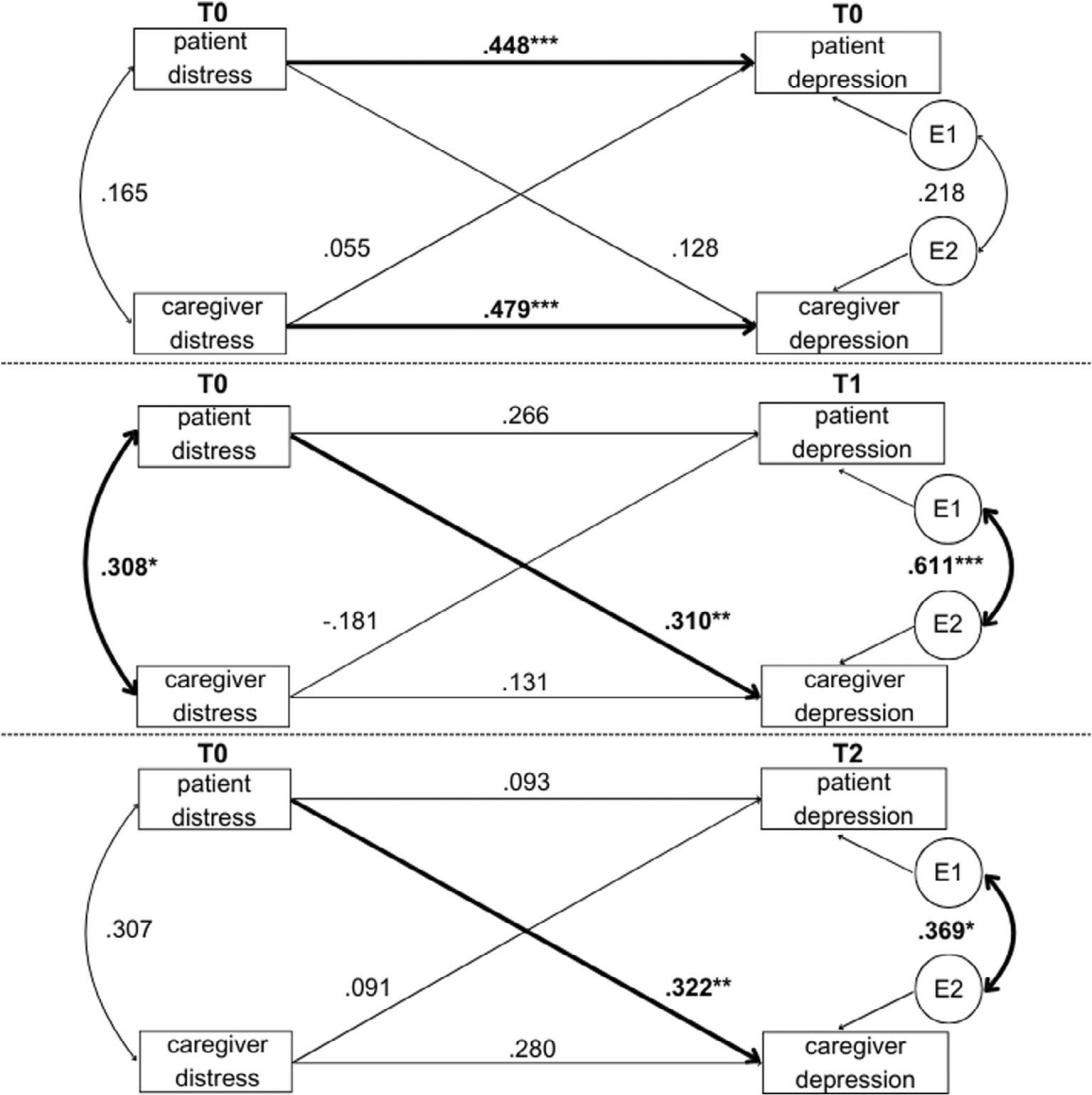
Actor partner interdependence models for depression over time. Values represent standardized beta coefficients. E1 = residual/error term for patient, E2 = residual/error term for caregiver. T0 = Baseline, T1 = 3 months post‐diagnosis, T2 = 6 months post‐diagnosis. Number of included dyads for each analysis: T0: *N* = 58 dyads, T1: *N* = 43 dyads, T2: *N* = 41 dyads. *p < 0.05, **p < 0.01, ***p < 0.001.

No significant partner effects on depression were found at T0. The patients' psychosocial distress scores did not influence the caregivers' depression scores (*p* = 0.183, *r* = 0.17), nor were caregivers' distress scores associated with patients' depression scores (*p* = 0.699, *r* = 0.05). At T1 and T2, significant partner effects were observed, with patients' distress scores at T0 significantly affecting caregivers' depression scores at T1 (*p* = 0.007, *r* = 0.38) and T2 (*p* = 0.005, *r* = 0.40). No significant association was identified between caregivers' distress scores at T0 and patients' anxiety scores at T1 (*p* = 0.357, *r* = −0.14) and T2 (*p* = 0.649, *r* = 0.07). Figure 3 presents a detailed overview of the model.

#### 3.4.2 Actor Effects

At T0, the individual's distress score was significantly associated with his/her own anxiety score for both patients and caregivers (patients: *p* < 0.001, *r* = 0.46; caregivers: *p* < 0.001, *r* = 0.47). At T1 and T2, no significant associations between the individual's distress score at T0 and their own depression scores were found (patients: T1: *p* = 0.080, *r* = 0.26; T2: *p* = 0.550, *r* = 0.09; caregivers: T1: *p* = 0.377, *r* = 0.13; T2: *p* = 0.059, *r* = 0.28).

## 4 Discussion

The aim of this study was to assess the longitudinal association between psychosocial distress in patients with brain tumors at diagnosis and anxiety and depression in their caregivers over time. We focused on the associations within the dyads of patients and their respective caregivers using the APIM, taking into account the interrelationship of stress and coping mechanisms in these dyads [38].

Most notably, and in line with our hypothesis, patient psychosocial distress at diagnosis was associated with caregiver depression at both 3 and 6 months post‐diagnosis and with caregiver anxiety at 6 months post‐diagnosis. These findings are consistent with previous research suggesting that the emotional well‐being of caregivers is strongly influenced by the distress of cancer patients, highlighting the need to address the patient‐caregiver dyad as a unit of care (e.g., [22, 39]). For brain tumors, caregiver distress has been shown to be influenced by the patients' depression, age, and the functional status of the patient [25, 28]. Previous cross‐sectional findings in patients with pancreatic cancer showed that patient distress predicted caregiver anxiety and depression using APIM [22]. A study of dyads of patients with primary brain tumors and their caregivers, also using the APIM, provided the first evidence of dyadic effects related to fear of cancer recurrence [23]. However, in our study, patient psychosocial distress at diagnosis was not associated with caregiver anxiety at 3 months post‐diagnosis. In addition, no partner effects at diagnosis were observed. Since members of a dyad tend to influence one another progressively over time, this interrelation may not yet be evident at the initial stage of diagnosis. Our findings extend previous results, as we included longitudinal data allowing us to observe time‐related trends, especially related to disease progression. Furthermore, in contrast to other studies, our study was homogenous with regard to time since diagnosis [22, 23, 28].

Caregiver psychosocial distress at diagnosis did not predict subsequent patient depression or anxiety at any time point, suggesting an asymmetry in how the emotional states of patients and caregivers influence each other in the dyads studied. To our best knowledge, no study has yet assessed the longitudinal interrelationship of psychosocial burden in both patients with brain tumors and their caregivers [13, 40, 41]. In a cross‐sectional study of lung and gastrointestinal patient‐caregiver dyads, partner effects were found in both directions, with the interrelationship between patient and caregiver distress being influenced by the quality of dyadic coping [42]. As this patient population and their respective caregivers face specific challenges when compared to other cancer entities, such as a poor prognosis, neurocognitive and personality changes, the interaction pattern between patient and caregiver psychosocial outcomes might differ from other populations [43, 44]. Additionally, a review from Chen et al. [45], which summarized the study results of various cancer entities (e.g., prostate or breast cancer), suggests that patients may benefit more directly from dyadic coping strategies, whereas caregivers are more likely to withhold or suppress their own distress, engage in protective buffering, or rely on supportive coping. Although these findings do not refer to brain tumor dyads, they may explain why caregiver psychosocial distress did not predict patient depression or anxiety in our sample and highlight asymmetries in emotional exchange and coping within patient‐caregiver dyads.

Previous studies in patients with cancer and caregivers have suggested that an individual's psychosocial distress is closely related to their levels of anxiety and depression [44, 46, 47]. In addition, Reblin and Small [13] demonstrated that early burden in caregivers of patients with primary brain tumors can predict their later psychological distress. This finding aligns with our results, as psychosocial distress at diagnosis was significantly associated with anxiety and depression both in cancer patients and in caregivers. Anxiety and depression can therefore be identified by distress screening, highlighting the importance of a two‐step screening process at diagnosis [48]. Distress screening using an ultra‐short screening instrument such as the NCCN Thermometer is often more time‐efficient and has been more widely implemented in clinical settings compared to anxiety and depression screening. However, because baseline distress was not predictive of depression at follow‐up time points in our study, regular and recurrent screening is essential to address evolving psychological needs.

### 4.1 Study Limitations

This study has several limitations. First, our sample of patients with brain tumors was heterogeneous in terms of prognostic status, as we included both patients with a first diagnosis of a primary brain tumor and patients with newly diagnosed cerebral metastases. As symptoms and severity of the disease can affect both patient and caregiver distress, results may vary depending on the type of brain tumor. Second, we asked patients to identify their primary caregivers, which resulted in the inclusion of caregivers with different relationships to the patient (e.g., partners, family members; see Table 1). In addition, a higher percentage of caregivers were women. Unfortunately, we were not able to document whether participants received psycho‐oncological care during the study course and therefore could not include this as a variable in our analysis. Another limitation is the high number of dropouts during the study course, partly due to recruitment during the Covid‐19 pandemic, resulting in a relatively small sample size. As patients who dropped out of the study between baseline (T0) and 6 months post‐diagnosis (T2) reported lower baseline anxiety compared to completers, our findings may be more representative of patients experiencing higher levels of anxiety, and may not be fully generalizable. The pandemic may also have influenced the participants' distress levels. Although our distress scores align with pre‐pandemic studies in brain tumor patients [49], suggesting that any potential pandemic‐related effect was unlikely to be large, it can however not be excluded. However, considering the long follow‐up period of 6 months, the analysis of 41 dyads provides novel insights into the longitudinal interrelationship of patient‐caregiver distress.

### 4.2 Clinical Implications

Our findings highlight the substantial interrelationship between patient distress and caregiver anxiety and depression over time. Patient psychosocial distress at diagnosis was found to be associated with caregiver anxiety and depression even 6 months later, highlighting the importance of providing psychosocial support to newly diagnosed brain tumor patients and their caregivers as early as possible to prevent negative impact for both. However, current evidence on psychological interventions for patients with brain tumors and caregivers remains limited [50]. Notably, patients with brain tumors themselves have expressed that joint therapy sessions would be particularly beneficial and reported a strong need for increased support for their caregivers [16, 51].

While we would recommend screening both patients and their caregivers for psychosocial burden, screening of caregivers presents logistical challenges in routine clinical care. Our results confirm that elevated patient distress at diagnosis should be regarded as a critical indicator of potential future caregiver burden. As screening for patient distress is already integrated into clinical care, our results could therefore inform targeted caregiver support strategies based on patient distress screening [20]. Nevertheless, it is important to acknowledge that caregivers experiencing high psychosocial burden may be overlooked if only patient distress is assessed. However, patient distress can offer an early indication of potential caregiver burden when caregiver screening is not possible or not yet implemented into standard care.

## 5 Conclusion

This study highlights that the psychosocial distress experienced by patients with brain tumors is significantly associated with anxiety and depression in their caregivers. Specifically, our findings indicate that patient psychosocial distress at diagnosis predicts caregiver depression at 3 months, and anxiety and depression at 3 and 6 months post‐diagnosis. Surprisingly, we found no association between caregiver psychosocial distress and patient‐reported anxiety or depression. While we recommend the psychosocial screening of both patients and their caregivers, the observed association between patient and caregiver psychosocial burden highlights the potential for identifying caregivers at risk through systematic patient distress screening. These findings underscore the critical need for early psychosocial support, as patient distress is related to caregiver burden. We recommend that future clinical trials explore the feasibility of integrating patient distress screening as a tool to identify caregivers in need of support. In addition, future trials should focus on developing and evaluating psychosocial interventions that are tailored to the needs of both patients with malignant brain tumors and their caregivers.

## Author Contributions

Conceptualization: A.K., A.‐M.K., M.R. Data curation: A.‐M.K. Formal analysis: A.K., A.‐M.K., R.S., M.R., M.K.K. Funding acquisition: A.K., M.R. Investigation: A.‐M.K., C.Q., M.R. Methodology: A.‐M.K., R.S., M.R., A.K. Project administration: A.K., A.‐M.K., C.Q., M.R. Resources: A.K., M.S., M.R. Supervision: A.K., M.R. Validation: A.K., M.R. Visualization: A.‐M.K., M.K.K. Writing – original draft: A.‐M.K., M.K.K., A.K. Writing – review and editing: A.K., A.‐M.K., C.Q., M.K.K., R.S., M.S., M.R. All authors approved the submitted version of the article and agreed to be accountable for all aspects of the work.

## Ethics Statement

This primary study was approved by the ethics committee of the medical faculty at Heinrich‐Heine University Duesseldorf (Ethic ID: 2018–338‐ProspDEuA). The study was conducted in accordance with legal and regulatory requirements, as well as the general principles set forth in the Declaration of Helsinki, §15 of the German Medical Association's professional code of conduct ‘Berufsordnung für Ärzte, BOÄ’, and the applicable data protection law. All participants were informed about the study design and gave written informed consent before being included in the study.

## Conflicts of Interest

The authors declare no conflicts of interest.

## Data Availability

The data that support the findings of this manuscript are available on request from the corresponding author. The data are not publicly available due to privacy or ethical restrictions.

## References

[1] Q. T. Ostrom , H. Gittleman , G. Truitt , A. Boscia , C. Kruchko , and J. S. Barnholtz‐Sloan , “CBTRUS Statistical Report: Primary Brain and Other Central Nervous System Tumors Diagnosed in the United States in 2011–2015,” Neuro‐Oncology 20, no. suppl_4 (2018): iv1–iv86. 30445539 10.1093/neuonc/noy131 PMC6129949

[2] K. Iyer , S. Saini , S. Bhadra , S. Kulavi , and J. Bandyopadhyay , “Precision Medicine Advancements in Glioblastoma: A Systematic Review,” Biomedicine 13, no. 2 (2023): 1–13. 10.37796/2211-8039.1403 PMC10627207 37937301

[3] E. Wong , L. Zhang , L. Rowbottom , et al., “Symptoms and Quality of Life in Patients With Brain Metastases Receiving Whole‐Brain Radiation Therapy,” Supportive Care in Cancer 24, no. 11 (2016): 4747–4759. 27358169 10.1007/s00520-016-3326-8

[4] S. Goebel , M. von Harscher , and H. M. Mehdorn , “Comorbid Mental Disorders and Psychosocial Distress in Patients With Brain Tumours and Their Spouses in the Early Treatment Phase,” Supportive Care in Cancer 19, no. 11 (2011): 1797–1805. 20953802 10.1007/s00520-010-1021-8

[5] T. E. Gofton , J. Graber , and A. Carver , “Identifying the Palliative Care Needs of Patients Living With Cerebral Tumors and Metastases: A Retrospective Analysis,” Journal of Neuro‐Oncology 108, no. 3 (2012): 527–534. 22467138 10.1007/s11060-012-0855-y

[6] S. R. Chandana , S. Movva , M. Arora , and T. Singh , “Primary Brain Tumors in Adults,” American Family Physician 77, no. 10 (2008): 1423–1430. 18533376

[7] J. R. Schubart , M. B. Kinzie , and E. Farace , “Caring for the Brain Tumor Patient: Family Caregiver Burden and Unmet Needs,” Neuro‐Oncology 10, no. 1 (2008): 61–72. 17993635 10.1215/15228517-2007-040 PMC2600839

[8] T. H. Au , C. Willis , M. Reblin , et al., “Caregiver Burden by Treatment and Clinical Characteristics of Patients With Glioblastoma,” Supportive Care in Cancer 30, no. 2 (2022): 1365–1375. 34510238 10.1007/s00520-021-06514-0 PMC8727395

[9] M. G. Saria , A. Nyamathi , L. R. Phillips , et al., “The Hidden Morbidity of Cancer: Burden in Caregivers of Patients With Brain Metastases,” Nursing Clinics of North America 52, no. 1 (2017): 159–178. 28189161 10.1016/j.cnur.2016.10.002 PMC10239524

[10] S. M. Aoun , K. Deas , D. Howting , and G. Lee , “Exploring the Support Needs of Family Caregivers of Patients With Brain Cancer Using the CSNAT: A Comparative Study With Other Cancer Groups,” PLoS One 10, no. 12 (2015): e0145106. 26679505 10.1371/journal.pone.0145106 PMC4682982

[11] Q. Li , L. Zhang , C. Chen , et al., “Caregiver Burden and Influencing Factors Among Family Caregivers of Patients With Glioma: A Cross‐Sectional Survey,” Journal of Clinical Neuroscience 96 (2022): 107–113. 34840093 10.1016/j.jocn.2021.11.012

[12] V. Gulino , L. Brunasso , C. Avallone , et al., “Caregivers' Perspective and Burden of the End‐of‐Life Phase of Patients With Glioblastoma: A Multicenter Retrospective Study,” World Neurosurgery 192 (2024): e49–e55. 39214291 10.1016/j.wneu.2024.08.114

[13] M. Reblin , B. Small , H. Jim , J. Weimer , and P. Sherwood , “Mediating Burden and Stress Over Time: Caregivers of Patients With Primary Brain Tumor,” Psycho‐Oncology 27, no. 2 (2018): 607–612. 28801927 10.1002/pon.4527 PMC10111220

[14] L. G. Gordon , K. M. D. Merollini , A. Lowe , and R. J. Chan , “A Systematic Review of Financial Toxicity Among Cancer Survivors: We Can't Pay the Co‐Pay,” Patient 10, no. 3 (2017): 295–309. 27798816 10.1007/s40271-016-0204-x

[15] A. Nikbakht Nasrabadi , S. Pahlevan Sharif , K. A. Allen , et al., “The Role of Socioeconomic Status in the Relationship Between Social Support and Burden Among Cancer Caregivers,” European Journal of Cancer Prevention 31, no. 2 (2022): 198–203. 33899748 10.1097/CEJ.0000000000000683

[16] A. R. Loughan , M. Reid , K. D. Willis , et al., “The Burden of a Brain Tumor: Guiding Patient Centric Care in Neuro‐Oncology,” Journal of Neuro‐Oncology 157, no. 3 (2022): 487–498. 35394618 10.1007/s11060-022-03993-x

[17] Leitlinienprogramm Onkologie , Psychoonkologische Diagnostik, Beratung und Behandlung von erwachsenen Krebspatient*innen, Langversion 2.1: AWMF‐Registernummer: 032‐051OL (2023), https://www.leitlinienprogramm‐onkologie.de/leitlinien/psychoonkologie/.

[18] M. B. Riba , K. A. Donovan , K. Ahmed , et al., “NCCN Guidelines Insights: Distress Management, Version 2.2023: Featured Updates to the NCCN Guidelines,” Journal of the National Comprehensive Cancer Network 21, no. 5 (2023): 450–457. 37156476 10.6004/jnccn.2023.0026

[19] A. J. Applebaum , E. Schofield , A. Kastrinos , et al., “A Randomized Controlled Trial of a Distress Screening, Consultation, and Targeted Referral System for Family Caregivers in Oncologic Care,” Psycho‐Oncology 33, no. 2 (2024): e6301. 38363002 10.1002/pon.6301 PMC11250988

[20] M. Suresh , R. Risbud , M. I. Patel , et al., “Clinic‐Based Assessment and Support for Family Caregivers of Patients With Cancer: Results of a Feasibility Study,” Cancer Care Research Online 3, no. 4 (2023): e047. 38328267 10.1097/cr9.0000000000000047 PMC10846853

[21] L. N. Bialon and S. Coke , “A Study on Caregiver Burden: Stressors, Challenges, and Possible Solutions,” American Journal of Hospice & Palliative Care 29, no. 3 (2012): 210–218. 21803785 10.1177/1049909111416494

[22] B. T. Xia , A. K. Otto , K. Allenson , et al., “Patient‐Caregiver Dyads in Pancreatic Cancer: Identification of Patient and Caregiver Factors Associated With Caregiver Well‐Being,” Journal of Behavioral Medicine 45, no. 6 (2022): 935–946. 35986871 10.1007/s10865-022-00354-x

[23] S. E. Braun , F. J. Aslanzadeh , L. Thacker , and A. R. Loughan , “Examining Fear of Cancer Recurrence in Primary Brain Tumor Patients and Their Caregivers Using the Actor‐Partner Interdependence Model,” Psycho‐Oncology 30, no. 7 (2021): 1120–1128. 33599334 10.1002/pon.5659 PMC10440852

[24] G. Bodenmann , “Dyadic Coping‐a Systematic‐Transactional View of Stress and Coping Among Couples: Theory and Empirical Findings,” European Review of Applied Psychology 47 (1997): 137–140.

[25] D. A. Forst , A. F. Podgurski , K. M. Quain , et al., “Factors Associated With Psychological Distress in Caregivers of Patients With Malignant Gliomas,” Supportive Care in Cancer 30, no. 7 (2022): 5811–5820. 35353218 10.1007/s00520-022-06989-5

[26] A. Karger , A.‐M. Kisić , C. Quente , et al., “Longitudinal Psychological Distress After Malignant Brain Tumor Diagnosis: A Multilevel Analysis of Patients and Their Caregivers,” Psycho‐Oncology 34, no. 1 (2025): e70064. 39794295 10.1002/pon.70064 PMC11723856

[27] E. von Elm , D. G. Altman , M. Egger , et al., “The Strengthening the Reporting of Observational Studies in Epidemiology (STROBE) Statement: Guidelines for Reporting Observational Studies,” International Journal of Surgery 12, no. 12 (2014): 1495–1499. 25046131 10.1016/j.ijsu.2014.07.013

[28] C. Y. Finocchiaro , A. Petruzzi , E. Lamperti , et al., “The Burden of Brain Tumor: A Single‐Institution Study on Psychological Patterns in Caregivers,” Journal of Neuro‐Oncology 107, no. 1 (2012): 175–181. 21968946 10.1007/s11060-011-0726-y

[29] A. Mehnert , D. Müller , C. Lehmann , and U. Koch , “Die deutsche Version des NCCN Distress‐Thermometers: Empirische Prüfung eines Screening‐Instruments zur Erfassung psychosozialer Belastung bei Krebspatienten,” Zeitschrift für Psychiatrie, Psychologie und Psychotherapie 54, no. 3 (2006): 213–223.

[30] C. Herrmann‐Lingen , U. Buss , and R. P. Snaith , Hospital Anxiety and Depression Scale: HADS‐D; Deutsche Version: Manual (Huber, 2011).

[31] I. Bjelland , A. A. Dahl , T. T. Haug , and D. Neckelmann , “The Validity of the Hospital Anxiety and Depression Scale. An Updated Literature Review,” Journal of Psychosomatic Research 52, no. 2 (2002): 69–77. 11832252 10.1016/s0022-3999(01)00296-3

[32] A. Hinz , P. Y. Herzberg , F. Lordick , et al., “Age and Gender Differences in Anxiety and Depression in Cancer Patients Compared With the General Population,” European Journal of Cancer Care 28, no. 5 (2019): e13129. 31290218 10.1111/ecc.13129

[33] M. M. Oken , R. H. Creech , D. C. Tormey , et al., “Toxicity and Response Criteria of the Eastern Cooperative Oncology Group,” American Journal of Clinical Oncology 5, no. 6 (1982): 649–656. 7165009

[34] W. L. Cook and D. A. Kenny , “The Actor–Partner Interdependence Model: A Model of Bidirectional Effects in Developmental Studies,” International Journal of Behavioral Development 29, no. 2 (2005): 101–109.

[35] D. A. Kashy and D. A. Kenny , “The Analysis of Data From Dyads and Groups,” in Handbook of Research Methods in Social and Personality Psychology, ed. H. T. Reis and C. M. Judd (Cambridge University Press, 2000), 451–477.

[36] Y. Rosseel , “Lavaan: An R Package for Structural Equation Modeling,” Journal of Statistical Software 48, no. 2 (2012): 1–36.

[37] R Core Team , R: A Language and Environment for Statistical Computing (R Foundation for Statistical Computing, 2023), https://www.R‐project.org/.

[38] K. Kayser , L. E. Watson , and J. T. Andrade , “Cancer as a ‘We‐Disease’: Examining the Process of Coping From a Relational Perspective,” Families, Systems & Health 25, no. 4 (2007): 404–418.

[39] K. R. Ellis , S. Oh , H. K. Hecht , and L. Northouse , “Symptom Distress and Quality of Life Among Black Americans With Cancer and Their Family Caregivers,” Psycho‐Oncology 30, no. 8 (2021): 1356–1365. 33861891 10.1002/pon.5691 PMC8672379

[40] P. R. Sherwood , B. A. Given , C. W. Given , et al., “Predictors of Distress in Caregivers of Persons With a Primary Malignant Brain Tumor,” Research in Nursing & Health 29, no. 2 (2006): 105–120. 16532486 10.1002/nur.20116

[41] A. Hricik , H. Donovan , S. E. Bradley , et al., “Changes in Caregiver Perceptions Over Time in Response to Providing Care for a Loved One With a Primary Malignant Brain Tumor,” Oncology Nursing Forum 38, no. 2 (2011): 149–155. 21356653 10.1188/11.ONF.149-155 PMC3880557

[42] G. Rapelli , S. Donato , A. F. Pagani , et al., “The Association Between Cardiac Illness‐Related Distress and Partner Support: The Moderating Role of Dyadic Coping,” Frontiers in Psychology 12 (2021): 624095. 33679540 10.3389/fpsyg.2021.624095 PMC7925924

[43] R. Caruso , M. G. Nanni , M. B. Riba , S. Sabato , and L. Grassi , “The Burden of Psychosocial Morbidity Related to Cancer: Patient and Family Issues,” International Review of Psychiatry 29, no. 5 (2017): 389–402. 28753076 10.1080/09540261.2017.1288090

[44] T. L. Deshields , Y. Asvat , A. R. Tippey , and J. R. Vanderlan , “Distress, Depression, Anxiety, and Resilience in Patients With Cancer and Caregivers,” Health Psychology 41, no. 4 (2022): 246–255. 35324246 10.1037/hea0001170

[45] M. Chen , J. Gong , Q. Cao , X. Luo , J. Li , and Q. Li , “A Literature Review of the Relationship Between Dyadic Coping and Dyadic Outcomes in Cancer Couples,” European Journal of Oncology Nursing 54 (2021): 102035. 34520996 10.1016/j.ejon.2021.102035

[46] C. G. Ng , S. Mohamed , K. Kaur , A. H. Sulaiman , N. Z. Zainal , and N. A. Taib , “Perceived Distress and Its Association With Depression and Anxiety in Breast Cancer Patients,” PLoS One 12, no. 3 (2017): e0172975. 28296921 10.1371/journal.pone.0172975 PMC5351853

[47] A. Gonzalez‐Ling , O. Galindo Vázquez , M. Espinoza Bello , et al., “Quality of Life, Anxiety, Depression, and Distress in Patients With Advanced and Metastatic Lung Cancer,” Palliative & Supportive Care 21, no. 4 (2023): 608–615. 36210754 10.1017/S147895152200116X

[48] L. Grassi , R. Caruso , M. B. Riba , et al., “Anxiety and Depression in Adult Cancer Patients: ESMO Clinical Practice Guideline,” ESMO Open 8, no. 2 (2023): 101155. 37087199 10.1016/j.esmoop.2023.101155 PMC10163167

[49] S. Goebel , A. M. Stark , L. Kaup , M. von Harscher , and H. M. Mehdorn , “Distress in Patients With Newly Diagnosed Brain Tumours,” Psycho‐Oncology 20, no. 6 (2011): 623–630. 21449043 10.1002/pon.1958

[50] A. Pace , L. Dirven , J. A. F. Koekkoek , et al., “European Association for Neuro‐Oncology (EANO) Guidelines for Palliative Care in Adults With Glioma,” Lancet Oncology 18, no. 6 (2017): e330–e340. 28593859 10.1016/S1470-2045(17)30345-5

[51] K. D. Willis , M. P. Reid , A. Fox , C. S. Kleva , P. Sherwood , and A. R. Loughan , “The Impact of a Primary Brain Tumor Diagnosis on Caregivers: Insights From the Patients' Perspective,” Supportive Care in Cancer 32, no. 9 (2024): 595. 39160352 10.1007/s00520-024-08783-x PMC11333512

